# Towards a Framework for High-Performance Simulation of Livestock Disease Outbreak: A Case Study of Spread of African Swine Fever in Vietnam

**DOI:** 10.3390/ani11092743

**Published:** 2021-09-19

**Authors:** Linh Manh Pham, Nikos Parlavantzas, Huy-Ham Le, Quang Hung Bui

**Affiliations:** 1 University of Engineering and Technology, Vietnam National University, 144 Xuan Thuy, Cau Giay, Hanoi 10000, Vietnam; lhham@agi.ac.vn (H.-H.L.); hungbq@vnu.edu.vn (Q.H.B.); 2 Campus Universitaire de Beaulieu, Université de Rennes, Inria, CNRS, IRISA, 35042 Rennes, France; nikos.parlavantzas@irisa.fr; 3 Agricultural Genetics Institute, Pham Van Dong, Bac Tu Liem, Hanoi 10000, Vietnam

**Keywords:** veterinary epidemiology, African swine fever, high-performance simulation, modeling, transmission and spread

## Abstract

**Simple Summary:**

Disease transmission simulation programs in veterinary epidemiology in general and in simulation of African swine fever in particular are often very diverse and require great computing power. However, such programs often share similar workflows from processing input/output data, performing simulations, or storing data. Our paper proposes a common architectural framework for livestock disease transmission simulation programs in order to both improve simulation performance and reduce the effort of developing new simulation programs. Our framework was evaluated with a simulation program of African swine fever transmission currently raging in Vietnam and some other countries around the world. The results from the evaluation experiments not only demonstrate the effectiveness of the framework in terms of performance but also have practical consulting value for decision makers in Vietnam and for international colleagues.

**Abstract:**

The spread of disease in livestock is an important research topic of veterinary epidemiology because it provides warnings or advice to organizations responsible for the protection of animal health in particular and public health in general. Disease transmission simulation programs are often deployed with different species, disease types, or epidemiological models, and each research team manages its own set of parameters relevant to their target diseases and concerns, resulting in limited cooperation and reuse of research results. Furthermore, these simulation and decision support tools often require a large amount of computational power, especially for models involving tens of thousands of herds with millions of individuals spread over a large geographical area such as a region or a country. It is a matter of fact that epidemic simulation programs are often heterogeneous, but they often share some common workflows including processing of input data and execution of simulation, as well as storage, analysis, and visualization of results. In this article, we propose a novel architectural framework for simultaneously deploying any epidemic simulation program both on premises and on the cloud to improve performance and scalability. We also conduct some experiments to evaluate the proposed architectural framework on some aspects when applying it to simulate the spread of African swine fever in Vietnam.

## 1. Introduction

The spread of diseases in livestock is an important research topic in veterinary epidemiology in order to provide warnings or advice to regulatory bodies responsible for the protection of public health in general and animal health in particular in terms of trends in the spread of diseases in herds [1]. As an essential and direct source of nutrition for humans, some diseases from livestock can infect humans if there is no timely intervention policy. Current common diseases, such as African swine fever (ASF) in even-toed ungulates, are a concern of many countries. In Vietnam, within a year of its appearance, ASF alone has spread to 63 provinces and cities, causing about 6 million pigs to be culled.

The study of applying computer technologies to decision making on disease control in livestock has been conducted by many research groups around the world, mainly focusing on building toolkits to identify characteristic epidemiological features [2,3,4] (e.g., use of radio-frequency identification (RFID) tags, surveillance cameras, or infrared thermometers) or programs that simulate the direction and extent of spread [5,6,7,8] of each disease on each type of livestock. In the second vein, the study of infectious disease transmission from a computational science perspective often occurs at the following three levels: (1) modeling of virus reproduction and deformation (modeling shape-shifting viruses) [9,10]; (2) modeling the immune system focuses on human or animal subjects [11,12,13]; (3) modeling disease spread developed for nearly a century, investigating macro factors such as inter-ethnic spread division of the population (city, district, and region) within a country and between countries [14,15,16]. At the third level, studies could delve into the analysis of patterns of within-herd spread between individuals in a herd mainly due to contact, eating habits, sanitary conditions of barns [17,18,19], or between-herd spread between farms mainly due to trade, grazing practices, or seasonal movement of herds [20,21]. Utilization of simulation models to assess outbreak outcomes and to determine cost-effective control methods, such as mobility control, vaccination, and depopulation is an essential mean for policymakers [22,23]. A stochastic compartmental model for the spread of Mycobacterium avium subspecies paratuberculosis (Map) into a confined dairy herd has been created by Marcé et al. [18]. The model represents the process of Map infection and management of herds. Despite the many well-known transmission pathways in conjunction with sanitary measures, the best method of controlling is to restrict the exposure of calves to adult excrement. The first spatio-temporal model of population and the dynamic of infection and indirect local transmission in dairy farms as well as animal transmission between farms has been established by Beaunée et al. [20]. Its consequences are that, for farms purchasing more than three animals annually, there is a rapid high risk of infection. Without appropriate management methods, even in places with few infected farms, the map propagation will not disappear naturally. In order to simulate bovine-viral diarrhea virus dissemination (BVDV) via a cow-calf herd and to evaluate the effect of the virus on the cattle herd such as abortion, calf morbidity, and calf mortality, a stochastic SIR model has been designed [24]. This paper indicated that both the median and 95 percent forecast interval for the range of effects of BVDV had the greatest decrease due to the combination of adult vaccination and calf testing and culling. Francis et al. [22] presented a summary of the modeling of cattle transmitted diseases by using the North American Animal Disease Spread Model (NAADSM). The impact of various connection architectures on the disease transmission in animals has been shown to have an important influence on epidemic speed and the number of infected herds. In March and early April 2001, a geographical simulation model was used to examine various management approaches for the Great Britain pandemic of foot-and-mouth disease [23]. This model anticipated an outbreak of around 1800 to 1900 farms and estimated that, between July and October 2001, the disease will be eliminated. This strategy comprised the slaughtering in 24 h of infected farms, killing of about 1 to 3 neighbor farms per infected farm, and the reduced inter-farm migrations of vulnerable animals. A simulation model was developed in order to investigate the development of the African swine fever within a pig unit and its size effect on ASF spread [25]. An animal can be susceptible, latent, subclinical, clinical, or recovered in the model. The results indicated that ASF propagation depends on the infection of subclinical animals and the residual of deceased animals, the viral transfer rate, and above all the unit size. Barongo et al. [26] provides a stochastic model to predict the dynamics of ASF transmission in a free-ranging pig population under a variety of intervention scenarios. The model provides information that biosecurity measures applied within 14 days of a pig pandemic might prevent up to 74% of pig fatalities as a result of ASF. Moreover, hypothetical vaccinations conferring 70% protection could save 65% of pig deaths if they are deployed before day 14.

While the benefits in terms of assisting policymakers in designing disease surveillance and control strategies are substantial, the simulation and decision support tools often face some computational power limitations, especially with input data sets that may involve tens of thousands of herds with millions of individuals distributed over a large geographical area such as a region or a country. For example, simulations of the transmission of bovine viral diarrhea in dairy cows or gastroenteritis due to the ruminant bacteria (*Mycobacterium avium* subsp. paratuberculosis) conducted on clusters at Oniris Nantes take days or even weeks to produce simulation results that have practical advice value [27]. Moreover, not all research groups can invest and maintain such high-performance but expensive systems, especially for only studying individual diseases. The second issue is the heterogeneity and non-standardization of the input data model because each research group manages its own set of parameters that are appropriate for its target disease and research concerns. Moreover, the data can come from many sources and are mainly raw data that have not been properly formatted or even handwritten data that have not been digitized. Another challenge is that sharing of data and work results between research groups is not performed frequently since epidemiological models are difficult to build or, once built, they are difficult to change. Simulation programs often only work independently without any combination on a single common platform to serve more accurate decision making.

In order to partially solve these challenges, the article introduces a novel framework that can be used for executing many different programs of livestock disease simulation at the same time, with new features offered in the following framework modules:

- Data Model Standardization is a module for transforming data related to livestock, veterinary epidemiology, etc., from many sources with different formats into uniform data models stored in tables of standardized database. These data models are designed according to the standards of each continent or region, for example, the SIGMA standard of European Food Safety Authority (EFSA), a standard for animal disease input data [28].
- Simulation Programs Management is a module for managing and distributing resources on premises as well as on the cloud for automatic and high-performance execution of simulations and centralized collection of outputs. This module also makes it possible to integrate different epidemic simulators on the same platform that manages and distributes resources for simulation computation, regardless of whether using a mathematical [16] or an agent-based model [29]. Moreover, a special new feature is that these programs can be allocated resources for operating and producing results at the same time.
- Analysis and Visualization is a module for handling data analysis on the output results stored centrally in the form of files or in the databases and is responsible for displaying the results in the form of tables, charts, histograms, or epidemiological maps using popular data representation programs in epidemiology, such as QGIS [30] or Epi Info [31], or in the web forms of cloud services.

The paper is structured as follows: Section 2 introduces our proposed architecture in detail and discusses software selection for each module. Section 3 presents a case study of African swine fever, which is a livestock disease spreading in many countries around the world including Vietnam. Section 4 describes various experiments performed in order to validate the framework using the ASF simulation model. Section 5 summarizes previous work performed in the field. Finally, Section 6 concludes the paper and guides future work.

## 2. Architecture

**Figure 1:**
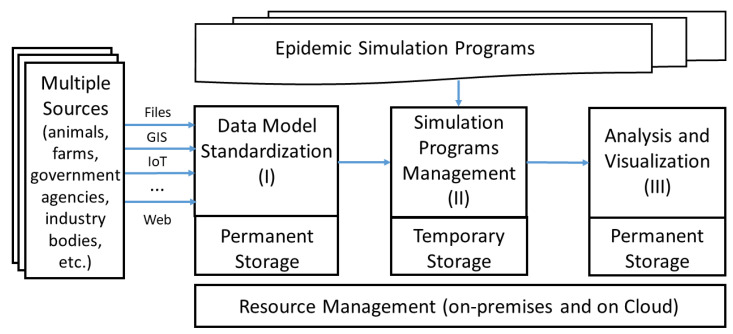
Common architecture for the management and execution of disease outbreak simulation programs in livestock.

Our proposed framework is illustrated in Figure 1. We will discuss each module in turn and the responsibilities it has to shoulder.

### 2.1. Data Model Standardization

A program that simulates the spread of disease in the herds, whether using synthetic or real data, may have to take input from multiple sources. These data sources are diverse and can include private data directly collected on the farm, traditional archives of local authorities, publicly available data on the internet, data collected from Internet of Things (IoT) sensors, and location data from a global geographic information system (GIS). These data sources, in addition to having different ownership rights, are not identical in terms of data format and description (metadata). Current decision-supported epidemic simulation tools are underutilizing these data sources. Using interoperable data standards and incorporating both public and private/copyrighted data can improve the overall advisory value of data and enhance our ability to share and reuse these data.

The number of data acquired by sensors has risen dramatically since the advent of digital agriculture [32]. Data streams may comprise sensor data gathered directly from animals, agricultural equipment or land surveillance locations, usually controlled and transmitted through wireless sensor networks. Additional agricultural data can be gathered via human observations, remote measurements, and daily farm records. Wireless sensors may be used to assess different animal conditions and behavior.

There are several main data sources used for veterinary disease management: from farm management information systems, from veterinary laboratories, or from veterinary clinics. On farms, automatic monitoring data of herd management and production quality indicators have been reported and reviewed in the article of Bartlett et al. [33]. However, no reports of the implementation of syndrome surveillance systems based on these data have been found. Mork et al. [34], by comparing data kept in farmers’ records with data reported by veterinarians and with the dairy industry’s cattle database in Sweden, showed that only 54% cases of disease in farmers’ livestock were treated by veterinarians. Even for the cases reported by both groups, the farmer kept more detailed and specific information than that reported by the veterinarian. The Bovine Syndromic Surveillance System (BOSS) system [35], although based on disease events, can be thought of as a system based on direct herd information, and it represents efforts that directly involve farmers. Theoretically, the underreporting rate should be low, as it targets sick populations of animals and not populations of animals receiving veterinary care. However, population coverage will be limited due to computer accessibility (and availability). This will become less and less of an issue, as more and more herds are managed with the help of computer systems. Increased use of computerized herd management tools can present a good opportunity for disease surveillance.

Veterinary laboratory testing requirements are one type of data of interest. They appear earlier than laboratory results and can be grouped into syndromes according to the nature of the disease and/or the symptoms observed by the veterinarian [36]. Stone investigated the potential of using laboratory test orders for syndrome surveillance in veterinary medicine and examined potential trends associated with this type of data [37]. The author also points to variation in annual filing rates and misclassification due to bias (veterinarians did not submit the correct samples or request the correct testing), but they still conclude that the data are consistent for syndrome surveillance. Laboratory test requests are often automated and recorded in digital form rather than clinical data [38]; thus, these data allow for a sustainable monitoring system to be built. Laboratories also represent a more centralized source of data, especially in animal medicine. However, their use depends on the data owner’s willingness to the sharing data.

In contrast to human medicine, in veterinary clinics at the time of service, in most cases, there is no requirement to transfer data to third party payers, such as insurance companies. This has resulted in veterinary clinic records being largely focused on client and billing management, and there is little incentive to develop and implement disease coding standards [39]. Despite these obstacles, the use of computer profiling is becoming standard practice in animal medicine, offering the opportunity to collect disease data. For example, the Small Animal Veterinary Surveillance Network (SAVSNET) [40], which intends to use real-time, fact-based data collection, will take advantage of the fact that around 20% of UK veterinary clinics use the same business management software. However, the lack of data standards in veterinary medicine means that data integration between clinics using different software remains an issue. The opportunities for data integration increase with the growth of enterprise veterinary operations [41]. Purdue University’s Banfield National Companion Animal Surveillance Program reported coverage of 2% of all dogs and cats in the United States; by using Banfield’s centralized database, the demographic information and medical information of a chain of veterinary hospitals widely available in the country are completely digitized [42]. The use of data from the same hospital network was also implemented by the LAHVA initiative [43].

There are several agricultural metadata standards, such as Agricultural Information Management Standards (AIMS) [44]. XML schemas such as AgXML [45] and AgroXML [46] address agriculture and arable farming, respectively. Over and above agriculture, the Dublin Core Metadata Initiative (DCMI) [47], which is crucial in view of the lack of agricultural standards, developed cross-domain and domain-specific metadata standards. However, an absence of interoperable data standards, especially applicable to veterinary epidemiology, requires further study in this field. The European Food Safety Authority recommends SIGMA, a standard for animal disease input data. The key entities involved in the SIGMA data model include Establishment, Sub Unit, Kept Animal, Geolocation, Disease Detection, and Monitoring/Surveillance Data. The development of the “Data Model Standardization” module, therefore, involves the development or use of a data model standardization tool that transforms the input data used by a simulation program to the data model specified in the standards, such as SIGMA. The standardized data will be stored in permanent storage for the purpose of reuse.

### 2.2. Simulation Programs Management

Epidemic simulation programs in livestock can use a variety of models, such as mathematical models, network-based models, or agent-based models. Each type of livestock can have simulations with different types of models using different programming languages (e.g., Java, C++, R, etc.) or different simulation platforms/tools (e.g., NetLogo [48], GAMA [49], NAADSM [50], etc.). What is noteworthy is that these programs can run simulations at many scales from the level of interactions between animals in the herd (within-herd) to between farms in areas or regions (between-herd). No matter what kind of model is used at any scale, given the wide variety of input sources and huge data, the execution of these simulation programs requires a large enough number of resources to maintain computation over a long time in order to provide reliable results. Unfortunately, meeting this resource requirement is often beyond the capacity of local veterinary departments. Furthermore, small research groups in veterinary epidemiology often lack the resources to invest in high-performance infrastructure for their research. Cloud computing delivers those resources for extremely limited and irregular use without using large computer resources. Furthermore, cloud resources might be used in addition to existing infrastructures for normal office work in the Department of Animal Husbandry and Veterinary Services or similar organizations. Many current cloud platforms support hybrid resource management both on premises and on the cloud, such as OpenStack, Apache CloudStack, etc. By using a hybrid cloud model (between on-premises and public cloud), important and highly confidential data will be stored in private local clouds when needed. The data transmitted and stored on the public cloud are encrypted, and only authorized users are permitted to access these data.

Another problem is that simulation often has to be repeated many times which requires a lot of labor and can be tedious, resulting in subjective errors from humans. Therefore, the “Simulation Programs Management” module needs to support both the automatic running of various types of epidemic simulation programs (including post-run results collection) and resource allocation management in order to improve the performance of the simulation. In order to achieve performance gains, the simulation computations must be broken down into specific tasks and distributed to compute nodes (i.e., workers) in different compute infrastructures at the same time either on premises (e.g., clusters) or on the cloud. The module also needs to support the execution of multiple model types at the same time, while ensuring the necessary independence/isolation between these models. A specialized platform that supports the integration and high-performance execution of many simulation programs such as OpenMole [51] or Repast HPC [52] is required for the implementation of this module.

In order to render the integration of epidemic simulation programs easier, we recommend that these programs should at least implement the following specialized modules:

- The population module: This module can describe the entire population or describe individual livestock (especially in agent-based models). The population needs to be generated at the beginning of each simulation run and assigned macro attributes such as quantity and composition or micro attributes such as age, gender, or medical history, etc.
- The contact module: There are many ways that diseases can be transmitted in livestock, either by direct contact (animal-to-animal and droplet spread) or by indirect contact (airborne transmission, contaminated objects, food and drinking water, etc.). At the farm level, indirect transmission can also be caused by trade, grazing practices, or seasonal movements of livestock. Contacts also have properties such as duration or intensity.
- The disease module: The task of this module is to deal with everything related to diseases. An agent-based model, for example, has individual-level information that may be divided into three parts: first, animal disease and health state; second, contact-causing state changes; and third, contact-independent state change (e.g., movement control, vaccination strategy, etc.). A disease might alter animals’ everyday habit, for example, making them lie still or stop eating. Animal death that is managed by the population module may result from the disease.

While running simulation models, it may be necessary to extract instantaneous information; thus, some temporary data can be saved in the temporary storage or memory of the workers and will be collected by the master immediately upon request. In particular, agent-based models can benefit from this functionality to show the simulation of disease transmission while the model is still running.

### 2.3. Analysis and Visualization

This module is responsible for analyzing the resulting data collected after running the simulation. There are two methods to collect the results: (1) Either a monitoring module periodically watches and logs the steps and states of the simulation components, or (2) the agents themselves are in charge of reporting all information of interest. Often, the information of interest of a disease transmission simulation is not only about the number of infected individuals but also about the state of each individual or how the herd evolves over time cycles. In this manner, we can observe how a part of the population is affected by changes in the pattern at certain time intervals (for example, the impact of a movement control strategy imposed on a farm type). The resulting data collected from multiple simulations can be used independently or in combination in order to provide valuable statistical information or warnings and forecasts for farm owners or policymakers. Some result analysis methods such as sensitivity analysis are also useful for determining the impact of specific parameters on the model to adjust the input parameters of the simulation, making the simulation model more dependable.

In terms of data visualization, simulation results can be exported as files for use by desktop epidemiological analysis and visualization programs such as QGIS or Epi Info. These results can also be displayed in web-based form as a SaaS service in the cloud. The visualization services can be directly accessed in order to obtain data in permanent storage for displaying static information after the simulation is finished or for displaying real-time dynamic information while the simulation is still running (e.g., for viewing disease transmission in agent-based models).

## 3. ASF Case Study and Simulation Model

Cattle and pigs are an essential and direct source of nutrition for humans, and some diseases from livestock can infect humans if there is no timely intervention policy. Current common disease such as African swine fever in even-toed ungulates is a concern of many countries. In Vietnam, within a month of its appearance (February 2019 in Hung Yen), ASF alone broke out in nearly 15 provinces and cities, causing more than tens of thousands of pigs to be culled. Since then, ASF has spread to over 7700 communes in 600 districts of 63 provinces and cities with the total number of pigs culled being approximately 6 million equivalents to over 230 thousand tons (accounting for about 20% of total pork production of the country). By May 2020, more than half of the provinces and cities had not recorded an epidemic for more than 30 days. Due to the topicality of this epidemic in Vietnam, we chose ASF as the focus of this article.

**Figure 2:**
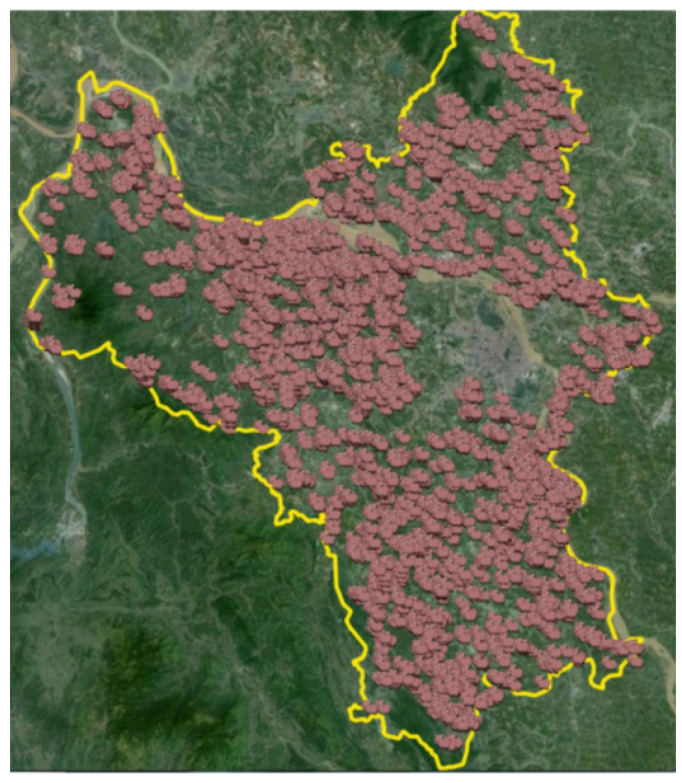
Geographical distribution of pig farms in Hanoi omitting the class of small farms (under 30 pigs) generated by QGIS.

We proceeded to build a model of the spread of ASF on all farms of Hanoi city, the capital of Vietnam with nearly 10 million people (2019). The total pig production of Hanoi is about 1.4 million (accounting for 4.7% of the whole country). The number of pigs of large farms outside residential areas is more than 700,000, accounting for more than 50% of the city’s total pig herd. The farm locations and characteristics are required to construct the ASF transmission models. At provincial level, the number of livestock smallholdings and farms is 131,756 (figures for 2021 from the Hanoi Department of Livestock and Veterinary Medicine). Since there are no specific farm locations, random points for all farms in every commune are created by QGIS (see Figure 2). The coordinates will subsequently be retrieved and imported into the GAMA and NetLogo simulation platforms. We excluded livestock smallholdings (i.e., farms with less than 10 pigs), and a total of 23,162 farms are, therefore, selected and utilized in the simulation. Those farms are divided into three categories following Article 21, Decree No. 13/2020 of the Vietnamese Government detailing the Law on Animal Husbandry: small (less than 30 pigs), medium (less than 300 pigs), and large (more than 300 pigs). The share of each sort of production is 78.8% (18,264), 19.6% (4532), and 1.6% (366), respectively.

In Vietnam, ASF is often transmitted by indirect contact due to vehicle/human movements as well as swill feeding. For this reason, the model used for the ASF simulation in this article is the model of the trade network of domestic pigs in Vietnam with the particularity that large farms are a direct source of supply to medium farms and do not directly supply to small farms. Medium farms will be a direct source of supply for small farms, and pigs from small farms are barely transferred to larger farms. This is consistent with the actual situation of pig production in Vietnam because a system for continuous flow (CF) is employed in small farms, meaning that pigs from various sources which are medium farms with uncertain infection status are regularly replaced. While at big and medium-sized farms where state of infection and superb biosecurity are known, the all-in-all-out (AIAO) method is followed, and those farms bring in fresh pigs in batches. These factors are used in our estimations of the infectious life for the three farm types in which big and medium-sized farms may stay infectious for a shorter period. Small farms, however, remain infectious throughout the entire simulation period because of the continual reintroduction of pigs.

In the ASF model used, there are three main parameters to consider: (1) mean contact rate per week (CR), (2) transmission probability (TP), and (3) contact distance between farms (CD). Details of these parameters will be discussed in the following section.

## 4. Evaluation

### 4.1. Settings of Model Parameters

We developed implementations of the ASF model mentioned in Section 3 by using both GAMA and NetLogo simulation platforms. We supposed that neither pig had ASF viral resistance. If one pig was infected it was deemed contagious across the farm. In the baseline scenario, a medium farm was infected, which is also set to an infected state in the next repeats. It is supposed that the remaining farms were susceptible to infection from the start and stayed in this state until the conclusion of the research duration for small-sized farms or until the end of a prearranged time for medium-sized and large-sized farms, then they are susceptible again (following SIRS model [53]). As a result, medium-sized and large-sized farms during the simulation might be infected several times.

Actual data from an agency survey on population, size of herds, pig farm health conditions, number of pigs, and interaction with other farms have been used to determine trustworthy parameters of the mean contact rates per week (CR) between various farms in Hanoi. The CR parameters with their Poisson distributions amongst various farm categories are shown in Table 1. TP parameters for both indirect and direct contacts are set to the same value for small-sized and medium-sized farms (0.5), but for big ones the value of indirect and direct contact is set at 0.005 and 0.5, respectively, owing to comparably high biosecurity standards [54]. We postulate that, according to the kind of farms, various biosafety levels determine the various durations of infection (small farms: 1 year; medium farms: 14 to 16 weeks; big farms: 6 weeks). For CD parameters, a PERT distribution of at least 0.5 km with the most likely values of 10 km and a maximum of 50 km was determined. This parameter is set based on the actual geographical conditions in Hanoi. In our implementations of ASF model, one GAMA cycle and one NetLogo tick are both set to 1 day. Both implementations are converted to OpenMole programs for running in distributed environments.

The simulation model is performed for one year, which is sufficient to encompass an entire cycle of pig production (6–8 months in Vietnam). Since ASF vaccination does not exist, we evaluate the efficiency of the restriction of movement in reducing contact rates for direct and indirect contacts by 20%, 40%, and 80%. It is assumed that movement constraints will be applied within 6 weeks following the pandemic announcement for the baseline scenario.

### 4.2. System Setup

**Figure 3:**
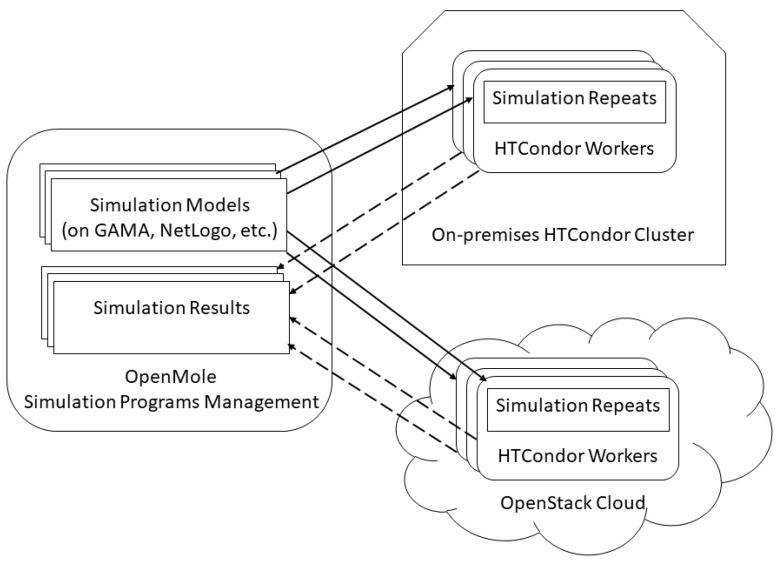
System architecture of proposed framework’s implementation.

We deployed a prototype of the proposed framework with open sources software components such as OpenStack for cloud resource management, HTCondor for on-premises cluster management, and OpenMole for simulation programs management. In terms of virtualization, result files are outputted in the formats supported by desktop-based programs such as QGIS and Epi Info. We also developed a web-based application that supports rendering of the epidemic simulation interface and graphs from GAMA [55]. While on-premises simulations are performed on HTCondor cluster, nodes serving computations for the simulation in the cloud environment are virtual machines (VMs) provisioned by OpenStack cloud installed in the data center for research at VNU University of Engineering and Technology (VNU-UET). The simulation environments are managed centrally by OpenMole so that compute resources of various environments can be distributed simultaneously. Figure 3 depicts our system architecture for the prototype.

### 4.3. Standardization of Animal Disease Data Model

**Figure 4:**
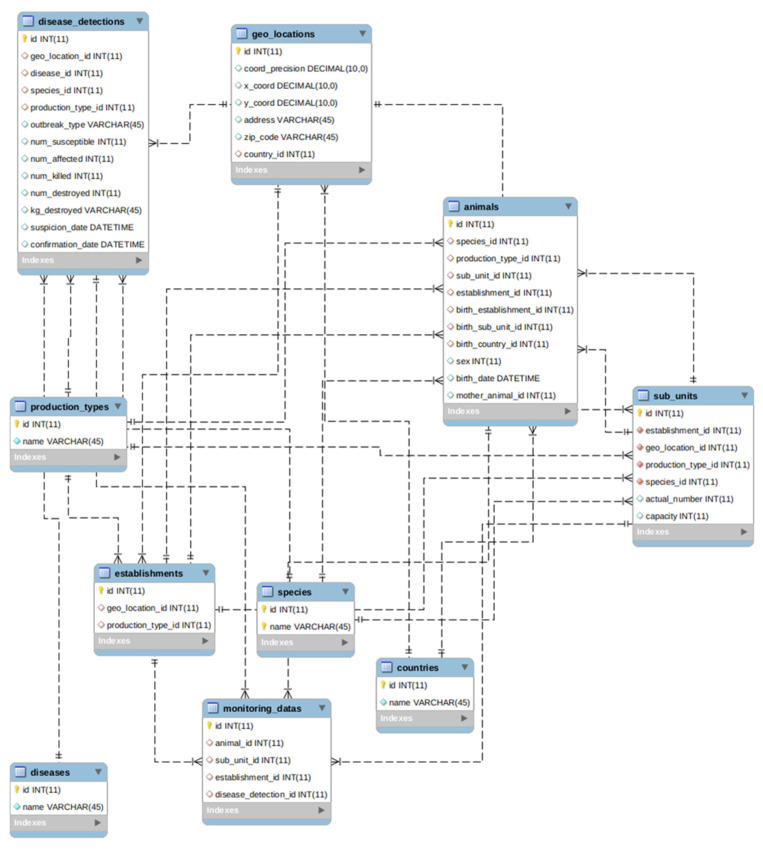
Entity Relation Diagram of our implementation of SIGMA animal disease data model.

As mentioned in Section 2.1, we selected SIGMA of EFSA as a standard of animal disease data model. This model captures all entities of livestock, which are data sources used in simulations of veterinary epidemiology. Our implementation of SIGMA is depicted in Figure 4 with some definitions as follows:

- Establishments: Any premises, buildings or any habitat or location where animals or germinal products are kept temporarily or permanently, excluding houses in which pets are kept as well as veterinary offices or clinics;
- Sub_units: Animals management group as part of an establishment such as flocks, pen, herds, houses, sheds, etc;
- Animals: Any terrestrial human-kept and registered animal that has a single identity number;
- Geo_locations: Positioning in the greatest possible resolution of the unit of interest, i.e., an establishment or a single animal;
- Disease_detections: Information string on probable epidemic reports, as documented in government veterinary agencies’ information system where accessible or in other public systems (e.g., EFSA DCF, and WAHIS);
- Production_types: Type of the establishment’s finished product or objective for which animals are maintained and/or raised;
- Species: The name, genus, species, and breed of the sub-unit of interest. This is especially significant in instances where the individual animals have no animal identification;
- Diseases: Disease to be reported;
- Countries: The ISO code of the country of birth or farm of the kept animal;
- Monitoring_data: Data from the surveillance or monitoring of kept animals, farms, diseases, and others.

In Vietnam, it is a fact that digitized data at the individual animal level or even at the livestock farm level is quite rare. Therefore, in the ASF model used for the experiments, some entities of the SIGMA data model are not used, especially the Animals entity where data about each individual animal are stored. However, for disease outbreak simulations in Europe or America where sources of data at individual animal level are well defined and collected, it is possible to standardize the data by using the Animals entity such as our previous study in the simulation of spread of bovine viral diarrhea virus (i.e., BVDV) on dairy cattle herds [56].

**Figure 5:**
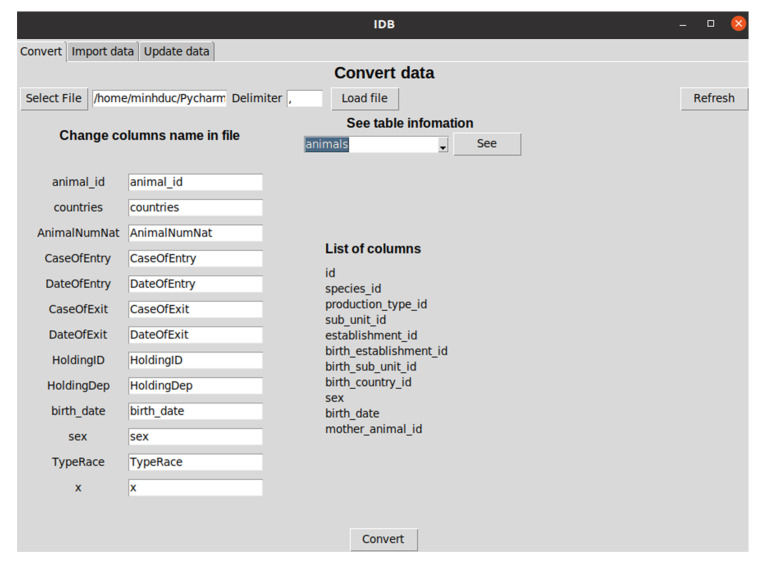
Graphical user interface of the SIGMA-based data model standardization software.

We developed an open-source software [57] helping users to transform any data models to the SIGMA one. A graphical user interface of the software is shown in Figure 5.

### 4.4. Performance Analysis

In this section, we perform a couple of experiments in order to evaluate the performance and functionality of the proposed framework when implementing the simulation of the ASF disease transmission model.

#### 4.4.1. Cost-Effective Assessment

With a realistic simulation of ASF outbreak, we assessed our proposed framework as a field trial in veterinary epidemiology. In each cycle (i.e., 1 day) of the ASF model, all farms participate in contacting the network and change the network together; thus, operations on farms must be executed sequentially, farm after farm. However different scenarios of the simulation as well as their repeats can be run independently and distributed to different local or remote processes for running in parallel.

The performance of running ASF simulation was compared in various environments using on-premises and cloud resources. With on-premises resources, the repeats are (I) conducted sequentially in a regular computer (Acer Nitro 5, Core i5 2.50 GHz, 8GB RAM, OS CentOS 7) where all four cores are allowed to be use for the simulation or (II) conducted in a cluster of the HTCondor batch system. In the (I) environment, our proposed framework is not used whereas OpenMole asks HTCondor to manage and distribute simulation jobs including repeats to compute nodes (i.e., workers) in the cluster of the (II) environment (see Figure 3). We deployed three Dell PowerEdge R740XD rack servers as HTCondor nodes with a specification of Intel Xeon Gold 2.4 GHz supporting up to 80 CPU cores, 256 GB RAM, and using OS CentOS 7. In order to ensure fairness in the number of CPU cores, only 4 out of 80 cores of each server are configured to be used. With cloud environment, HTCondor submits simulation jobs to three VMs in our private OpenStack cloud (III). Each VM is equipped with four vCPUs and 8GB RAM, also running CentOS 7. Additionally, a hybrid environment (IV) is made between the HTCondor cluster and OpenStack cloud, enabling jobs to be submitted to both systems.

OpenMole is responsible for measuring the execution time of the ASF simulation and its repeats. We implemented three parameter sets for three movement control strategies on reducing contact rates by 20%, 40%, and 80%. Each movement restriction also has three parameter sets for timing controls which are 3, 6, and 9 weeks of postponement of movement restriction after detection of outbreaks. Each of nine scenarios is repeated 50 times in order to obtain the statistical mean. One parallel simulation repeat is guaranteed to run in one core of participating nodes by OpenMole and HTCondor. Each repeat involves a calculation of 23,162 farms in every single 364 cycles. Firstly, the following formula is used to calculate the performance of each simulation in the environments from (I) to (IV).

(1) $$\mathrm{Performance}\;(P)=\frac{\mathrm{Number}\_\mathrm{of}\_\mathrm{repeats}\;\left(n\right)}{\mathrm{Execution}\_\mathrm{time}\;\left(\mathrm{hours}\right)}$$

We have utilized a modified version of Formula (1) to assess cost-effectiveness:

(2) $$P_{c}=\frac{\mathrm{Performance}}{\mathrm{Costs}}$$

where Costs are referenced from the similar c5.xlarge configuration (i.e., instance type) on Amazon EC2 [58]. For the on-premises nodes, a fixed initial infrastructure investment and IT system maintenance costs are added to the referenced unit cost [59]. Details on hardware configurations and costs of node types in the test environments are shown in Table 2.

Table 2 shows some performance results; the best performance on average was observed for the cluster environment (II), followed by the hybrid (IV), and cloud (III) environment. The on-premises environment with a standalone desktop (I) produced the worst performance. However, while the cost-effective performance was the best for the (III) environment, cluster one fell to third place. The hybrid one provides better cost-effective performance than the cluster one, and it is useful when the on-premises resources are limited. To add one more thing, the overall simulation duration in the cloud was around 9 h and 22 min. This simulation costs a total of USD 4.8 for three VMs. If six instances had been rented for 4 h and 41 min, the execution time would have been significantly lower for the same cost instead of operating three instances for 9 h and 22 min. This proves the effectiveness of our proposed framework’s approach when supporting simulation execution in different distributed environments at the same time.

#### 4.4.2. Scalability

We also conducted another experiment to evaluate the scalability of our framework on both shared and distributed memory architectures. We proceeded to simulate a medium-scale ASF propagation comprising of all 467 communes in Hanoi. This setup allows us to assess the scalability of the framework by increasing the number of processes progressively. We take the execution with four CPU cores for each node as base, both for the distributed and the shared configurations. With distributed configuration, we reuse the HTCondor cluster of three Dell PowerEdge R740XD rack servers. With shared configuration, we picked one out of three Dell PowerEdge R740XD servers and limited the number of CPU cores allowed to use for the simulation. The number of cores doubled until 32 in both configurations.

**Figure 6:**
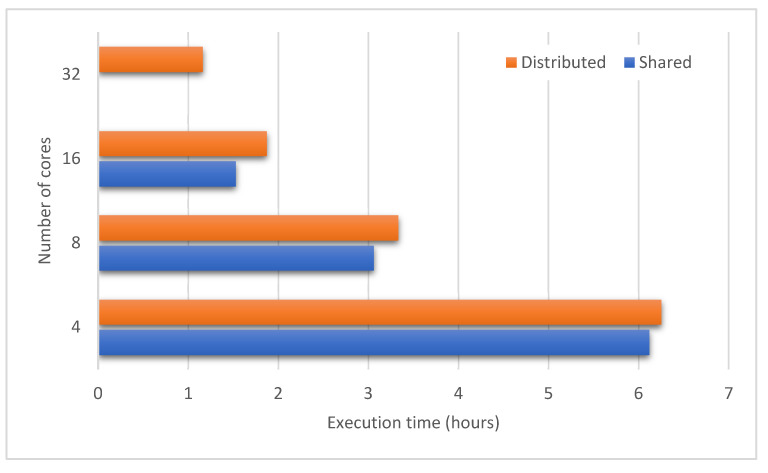
Execution time of the distributed and shared configurations when the number of CPU cores varies.

**Figure 7:**
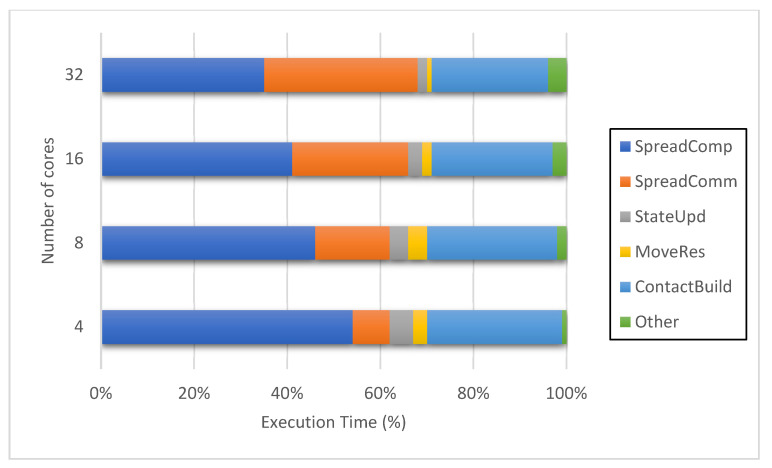
Execution time of simulation operations of the distributed configuration when the number of CPU cores varies.

Figure 6 demonstrates how the applications scale the performance of the base execution in both configurations. The distributed one scales up well to 32 cores per node in the cluster and the shared memory system scales practically linearly because of a minimal intra-node communication cost. The experiment is limited to 32 cores from the above in the shared memory system, since a maximum of 80 cores is provided to each node and 96 cores are not achievable. In order to further evaluate performance, we assess the time to complete each operation inside a single cycle of the distributed configuration, including contact network build (ContactBuild), spread computation (SpreadComp), spread communication (SpreadComm), movement restrictions (MoveRes), state update (StateUpd), and others. Figure 7 shows the proportion of the average time spent during all 364 cycles for each operation. In the case of simulation with additional processes, the proportion of total time taken for computation (SpreadComp and StateUpd) drops, and the total time spent for communication (SpreadComm, MoveRes and ContactBuild) grows. More than half of the execution time is spent on communication activities with 16 and 32 cores. The ContactBuild execution time is much longer than the SpreadComm and MoveRes execution times. Other operations include synchronizing all operating processes, which might raise communication costs between nodes.

#### 4.4.3. Process Distribution

We ran this experiment on the distributed memory systems such as the cluster, cloud, and hybrid environments mentioned in 4.4.1. Each simulation job includes a batch of processes which are simulation repeats. In the distributed environments, these processes are delivered to a pool of CPU cores according to a specific method. In the first method, the processes of repeats are distributed in a batch-based manner in which batches of a fixed number of processes are allocated to the compute nodes in the round. This distribution may cause difficulties if some repeats of batches performed in a node take longer than other nodes to complete. This results in some nodes always seeming busier than the other ones.

The second method provides more fine-grained batches in order to compute nodes. In fact, only one process is sent to the nodes in round-robin manner. The waiting time for long-running repeats is shortened, but the overhead for inter-process communication increases accordingly.

The third method uses our previous job-based algorithm introduced in [27]. The distribution of jobs of all processes involved in execution is taken into account. Initially, the algorithm allocates a higher number of repeats, which diminishes with the rest of the repeats. The objective of this approach is to have all nodes complete their computations at around the same time.

The percentage reduction for the total execution time of the round-robin and job-based distributions in comparison to the batch-based version is shown in Table 3. The simulation time in the cluster environment for batch-based distribution is around 6 h and 15 min for four processing batches. Each of the three distribution methods is assessed with each of the three-movement control strategies (i.e., 20%, 40%, and 80%). We can observe in every scenario that the job-based approach reduces the execution time the most.

## 5. Related Work

We spend this section mainly discussing similar studies in the development of architectures, platforms, and tools for performing high-performance simulations of disease outbreaks.

In order to complement pandemic simulations and leverage multi-core CPUs, Eriksson et al. [8] employed OpenMP. The assessment shows that performance may be enhanced with the varying computational load by dynamically switching between single and multi-core configurations. Parallelization at the distributed level is not considered in this study. EpiSimdemics, a highly scalable parallel code developed in Charm++, is introduced by Bhatele et al. [5] by employing agents-based modeling to simulate outbreaks of disease over enormous, realistic, and co-developing networks. The study describes an EpiSimdemics implementation modeling influenza propagation on several powerful computers. The authors contend that EpiSimdemics achieves five times greater speedup than the second fastest parallel code in the domain. The authors do not take parallelism into account at the distributed level, similarly to Eriksson et al. [8].

In order to increase performance, several solutions use parallelism at the distributed level. Perumalla and Seal [60] reported a reaction–diffusion simulation of epidemiological epidemics with optimistic, parallel, and discrete events execution. The simulation is at 65,536 cores, with acceleration exceeding 10,000, for a huge Cray XT5 system. The SEARUMS++ environment for ecological modeling was offered by Rao and Chernyakhovsky [6] in the context of the investigation of global avian influenza transmission. SEARUMS++ uses TimesWarp by employing parallel simulations on clusters synced in the environment. For complicated epidemic simulations, Bisset et al. [61] presented their modeling environment, known as Indemics, which is not confined to a particular disease model. While every prior solution may be scalable, it does not make use of the cloud’s advantages in cost reduction and resource efficiency.

Zou et al. [62] suggested the application of GPU clusters to execute large-scale epidemic simulations based on contact networks. This study discusses optimization approaches to improve memory access efficiency and minimize the latency of communication among compute nodes. Testing approaches on a cluster that has GPU computing nodes indicates that the execution on GPUs may be sped up 7.4 to 11.7 times compared to the CPU run. In order to speed up an influenza propagation agent-based simulation, Holvenstot et al. [7] utilized standard GPU devices. Experimental findings demonstrate that a GPU implementation is far quicker than a multi-threaded CPU implementation. Although previous systems showed GPU efficiency, they require buying costly, high-end hardware instead of clouds that now offer low-priced GPU-based resources.

A cloud-based framework for the simulation of epidemic disease spread has been proposed by Sukcharoen et al. [63]. The framework employs loop decomposition to parallelize the simulation on the Xen Cloud platform on many VMs in a private cloud. This framework is constrained to a particular epidemic model, which is a modified version of the SEIR model (susceptible, exposed, infectious, and recovered) that does not support implementation of hybrid environments. Price et al. [64] introduces a compute-intensive application methodology for cloud-based epidemic analysis. The method uses the Nimbus cloud to access on-demand resources. This paper does not explore the use of cloud resources for either performance or cost. Haris and Manzoor [65] presented cloud-based architecture to simulate dengue viral propagation in Pakistan. This architecture does not enable single-computer parallelization and does not take use of the elasticity of cloud resources.

To the best of our knowledge, our work is the first to propose a complete practical solution for building and executing high-performance simulations of livestock disease outbreaks on both on-premises and cloud environments at the same time. Moreover, the proposed framework is also the first one allowing the realization of any epidemic simulation model in livestock as well as combine simulation results from these models simultaneously.

## 6. Conclusions

Applying computing technology to simulate the spread of epidemic diseases presents multiple challenges. First, simulation data, models, and tools are typically diverse and tied to specific diseases and concerns of research groups, hindering research collaborations and decreasing the quality of decisions. Second, running epidemic simulations needs large amounts of computational power, practically requiring the combination of on-premises resources with cloud resources. Finally, storing data, managing simulation runs, and analyzing results can be complex and error-prone, requiring guidance and automated support.

To address these challenges, the paper proposed a novel framework for managing and executing epidemic simulation programs. The framework supports transforming heterogeneous data into uniform data models that are stored in standardized databases, thus facilitating data reuse. The framework also supports integrating different simulators and data analysis and visualization programs into a common platform, enabling sharing of models and tools, and facilitating decision making. Importantly, the framework includes automated support for simultaneously deploying simulation programs on various types of resources, both on-premises and cloud resources, in order to improve performance and reduce costs.

In order to evaluate the proposed framework, the paper described a prototype implementation and its application in simulating the spread of ASF in Vietnam. Based on this application, a number of experiments were performed. The experiments demonstrated that the framework supports simulation execution in different environments (cluster, cloud, and hybrid), enabling users to optimize performance and cost. They also demonstrated the scalability of the framework to large numbers of processors and the efficiency of its process distribution method. In future work, we plan to apply the framework to further case studies and perform a larger-scale evaluation of its usefulness and usability.

## Figures and Tables

**Table 1:** Movement structure of pig farming in Vietnam with mean direct/indirect contact rate per week following Poisson distributions in parentheses.

Farm Category	Small	Medium	Large
Small	Frequently (0.06/0.29)	Barely (-/0.29)	Barely (-/-)
Medium	Frequently (0.06/0.29)	Rarely (0.06/0.26)	Barely (-/3.2)
Large	Barely (-/-)	Rarely (0.06/0.26)	Barely (-/3.2)

**Table 2:** Configurations, costs, and performance of node types used in simulation tests.

Environment	I	II	III	IV
	On-Premises		Cloud	Hybrid
Platform	Acer Nitro 5	Dell PowerEdge R740XD	VM	II + III
CPU (core)	4	4/80	4	4
Memory (GB)	8	8/256	8	8
Cost (USD/hour)	0.15	0.67	0.17	0.42
Performance (P)	6	72	48	56
Cost-effective Performance (P_c_)	40	108	282	133

**Table 3:** Reduction in execution time in percentage of the round-robin and job-based distribution methods with respect to the batch-based one.

Compute Nodes	Distribution Policy	20%	40%	80%
1	Round-robin	7.32%	8.22%	7.62%
1	Job-based	12.90%	10.08%	9.00%
2	Round-robin	1.32%	4.68%	3.66%
2	Job-based	4.38%	7.68%	6.78%
3	Round-robin	3.54%	3.78%	3.66%
3	Job-based	8.04%	6.24%	5.40%

## Data Availability

The data presented in this study are available upon request from the corresponding author. The data are not publicly available due to confidentiality agreements.
